# Physician’s Burnout during the COVID-19 Pandemic: A Systematic Review and Meta-Analysis

**DOI:** 10.3390/ijerph20054598

**Published:** 2023-03-05

**Authors:** Abdulmajeed A. Alkhamees, Moath S. Aljohani, Simindokht Kalani, Amira Mohammed Ali, Fahad Almatham, Afnan Alwabili, Naif Abdullah Alsughier, Thomas Rutledge

**Affiliations:** 1 Department of Medicine, Unayzah College of Medicine and Medical Sciences, Qassim University, Unayzah 52571, Saudi Arabia; 2 Department of Family and Community Medicine, Unayzah College of Medicine and Medical Sciences, Qassim University, Unayzah 52571, Saudi Arabia; 3 Department of Psychology, Faculty of Education and Psychology, University of Isfahan, Isfahan 8174673441, Iran; 4 Department of Psychiatric Nursing and Mental Health, Faculty of Nursing, Alexandria University, Alexandria 5424041, Egypt; 5 VA San Diego Healthcare System, Department of Psychiatry, Psychology Service, University of California San Diego, San Diego, CA 92093, USA

**Keywords:** COVID-19, burnout, physicians

## Abstract

The burnout rate among physicians is expected to be higher during COVID-19 period due to the additional sources of physical and emotional stressors. Throughout the current COVID-19 pandemic, numerous studies have evaluated the impacts of COVID-19 on physicians’ burnout, but the reported results have been inconsistent. This current systematic review and meta-analysis aims to assess and estimate the epidemiology of burnout and the associated risk factors during the COVID-19 pandemic among physicians. A systematic search for studies targeting physicians’ burnout was conducted using PubMed, Scopus, ProQuest, Cochrane COVID-19 registry, and pre-print services (PsyArXiv and medRχiv) for English language studies published within the time period of 1 January 2020 to 1 September 2021. Search strategies resulted in 446 possible eligible studies. The titles and abstracts of these studies were screened, which resulted in 34 probable studies for inclusion, while 412 studies were excluded based on the predetermined inclusion criteria. These 34 studies went through a full-text screening for eligibility, which resulted in 30 studies being included in the final reviews and subsequent analyses. Among them, the prevalence of physicians’ burnout rate ranged from 6.0–99.8%. This wide variation could be due to the heterogeneity among burnout definitions, different applied assessment tools, and even cultural factors. Further studies may consider other factors when assessing burnout (e.g., the presence of a psychiatric disorders, other work-related and cultural factors). In conclusion, a consistent diagnostic indices for the assessment of burnout is required to enable consistent methods of scoring and interpretation.

## 1. Introduction

The COVID-19 pandemic has put tremendous pressure on the world, especially frontline health care workers such as physicians, many of whom were overwhelmed by the pressure of treating patients while protecting themself and their families from the infection. On 11 March 2021, the Medscape website reported more than 3300 physicians and other health care workers (HWs) on their list who have died from the COVID-19 pandemic [1]. Lack of personal protective equipment, work overload, poor infection control, exposure to infected patients, and pre-existing medical conditions were identified as some of the risk factors for increased stress among HWs during COVID-19 [2]. During the epidemic, physicians experienced other stressors that contribute to their burnout, such as fears of taking the infection home to their family, increased demands for childcare during increased work hours and school closures, concern about their organization’s support of their personal and family needs if they develop an infection, and lack of access to up-to-date information and communication [3,4].

In addition to these numerous physical and interpersonal stressors, physicians have been put under psychological pressure in navigating the health changes and coping with shortening supplies. For example, physicians and other health professionals had a higher level of anxiety and depression, somatization, and insomnia during the pandemic [5,6], all of these factors can predispose physicians to be overworked, unable to engage in coping behaviors and prone to burnout [7].

Burnout is a “state of mental fatigue resulting from a person’s professional life” and is also known as the syndrome of physical-mental strength [8]. Burnout is an emotional and passive reaction to chronic job stress, the core of which is the gradual depletion of one’s internal energy resources, which includes emotional exhaustion, depersonalization, and a reduced sense of professional accomplishment [9] Professional burnout, emotional exhaustion, and loss of satisfaction with patient care affect doctors at all stages of their career, from residency trainees to certified specialists [10].

The 2021 Medscape National Physician Burnout Report reported a burnout rate of about 42%, which remains similar to the 43% reported in 2020 and 46% in 2015 [11,12,13]. These numbers indicate that rates of burnout are startling high among physicians—endorsed by more than 4 in 10 physicians—and were high even before the pandemic.

Burnout is an emerging critical issue facing specialists and trainees in all disciplines of medicine [14] A systematic review from 2006 to 2018 among physicians practicing in 41 European countries (regardless of their specialty) showed that burnout prevalence rates ranged from 2.5% to 72.0% [15]. Burnout in doctors is linked to adverse patient outcomes, including higher rates of medical errors [16,17] and poorer quality of care [18]. It is also linked to negative outcomes for doctors, including substance abuse [19] and suicide [20].

A systematic review (*n* = 25) from 1 January 2016 to 31 January 2021 examined the issue of physician burnout related to electronic health record (HER) in the first year of the COVID-19 pandemic and before. Results showed that EHR administrative burden increased burnout. Specifically time spent on documentation and workflow leads to loss of autonomy, lack of work-life balance, lack of control over one’s schedule, cognitive fatigue, overall loss of autonomy, and lack of good relationships with colleagues [21]. Another systematic review of burnout among surgical trainees and surgeons during the COVID-19 pandemic (*n* = 29, between April 2020 and December 2021, most were published in 2020 and in the United States) found that burnout was shown to occur between 6.0% and 86.0%. The authors concluded that COVID-19 is associated with burnout in up to 1 in 2 surgical residents and treating surgeons [22]. Claponea et.al (2022) also showed in a systematic review (*n* = 35, 2020 and 2021) that overall burnout ranged from 14.7% to 90.4% [23].

In contrast, another systematic review examining the burnout of doctors in the era of COVID-19 showed that the introduction of COVID-19 has heightened existing challenges that physicians face such as increasing workload, which is directly correlated with increased burnout. However, exposure to COVID-19 does not necessarily correlate with increased burnout, and is an area that needs further research [24].

Because physicians are the frontline healthcare workers in responding to the COVID-19 outbreak, burnout rates among physicians could be expected to be higher due to the additional sources of physical and emotional stress during this period. If true, this finding could have important implications for physicians, who could be motivated to engage in treatment for burnout symptoms, and healthcare organizations, who may benefit by investing in resources to facilitate the identification and treatment of burnout among their physician employees. In the current systematic review and meta-analysis, Given the inconsistencies between previous systematic reviews we reviewed the epidemiology of burnout and the associated risk factor during the COVID-19 pandemic among physicians.

## 2. Methods

### 2.1. Inclusion and Exclusion Criteria

Inclusion criteria were as follow: Studies published in English in a Peer-reviewed journal only; Studies examining physician burnout and associated risk factors during the COVID-19 pandemic; Studies that used standardized and validated instruments to measure burnout. We excluded studies reporting results in total for health care workers and not separately for physicians. Also, we excluded case reports, qualitative studies, reviews, protocols, editorials, and letters to the editor. Furthermore, any studies that did not measure physician’s burnout as an outcome or that evaluated burnout without the use of validated instruments.

### 2.2. Search Strategy and Selection Criteria

In designing the literature search and the review protocol, we followed the Preferred Reporting Items for Systematic Reviews and Meta-Analyses (PRISMA) statement [25]. The records search was conducted with the assistance of a librarian experienced in the systematic review process.

A systematic search by two independent authors (A.K. and M.J.) using PubMed, Scopus, ProQuest, Cochrane COVID-19 registry, and pre-print services (PsyArXiv and medRxiv) was searched from 1 January 2020, to 1 September 1 2021, and we removed duplicates. Using the following strategy: (burnout OR “burned out” OR depersonalization or “emotional exhaustion” or burnout, professional [MESH] or emotional stress [MESH] or psychological stress [MESH] or stress, psychological [MESH] OR compassion fatigue [MESH]) AND (“attending physician” OR physician or physicians [MESH] OR doctor or medical staff, hospital [MESH] OR physicians, primary care [MESH] or osteopathic physician [MESH]) AND (COVID-19 [MESH] OR SARS-COV-2 [MESH] OR 2019-nCoV Infection [MESH]) Also, we searched the full reference lists of all selected articles for additional relevant publications and the first 11 pages of google scholar.

Initially, two independent (A.K. and M.J.) screened the titles and the abstract of the records and then reviewed the full article if needed; any disagreements were solved by discussion between the two authors and a third author (R.T.).

### 2.3. Data Extraction and Quality Assessment

We used structured forms to extract data from each study, including authors, country, gender, age, sample size, study design, sampling method, burnout assessment tool, response rate, data collection time, the publication (journal or pre-print service), physician characteristics (e.g., resident or attending), number of physicians with burnout, scores on burnout scales, factors associated with burnout and the level of analysis (univariate or multivariate).

A modified version of the Newcastle-Ottawa Scale was used to assess the quality of the studies included in the systematic review [26]. This scale is designed for the rating of nonrandomized studies and assesses quality in several domains: sample representativeness and size, comparability between respondents and nonrespondents, which is based on three broad perspectives, and the identification of either the exposure or outcome of study cohorts. Studies were judged to be at low risk of bias (≥3 points) or high risk of bias (<3 points). Disagreements were resolved by discussion and a third reviewer.

### 2.4. Statistical Analyses

We calculated aggregated meta-analysis results from studies using the DerSimonian and Laird method in a random-effects model, calculated with meta-analysis software [27]. The primary meta-analytic outcome consisted of burnout rates across physicians participating in the studies. We further planned a priori meta-regression analyses within the studies to explore anticipated heterogeneity in the burnout rates using study-level characteristics. These included: (a) study location (U.S. and Canada, Europe, Arab countries, South American, and Turkey and Pakistan); (b) burnout measure (Maslach *Burnout* Inventory [MBI], abbreviated Maslach *Burnout* Inventory, Copenhagen Burnout Inventory, Professional Fulfillment Index); (c) period of data collection (1st half of 2020, 2nd half of 2020, 1st half of 2021); and (d) physician category (attending, residents/junior physicians, both). Meta-regressions were performed only with studies providing information regarding each characteristic. We quantified study heterogeneity using the I^2^ statistic [28], describing the percentage of total variation across studies that is caused by heterogeneity rather than chance. An I^2^ value of 25% is conventionally interpreted to indicate low heterogeneity; 50%, moderate heterogeneity; and 75%, high heterogeneity.

## 3. Results

### 3.1. Characteristics of Studies

**Figure 1:**
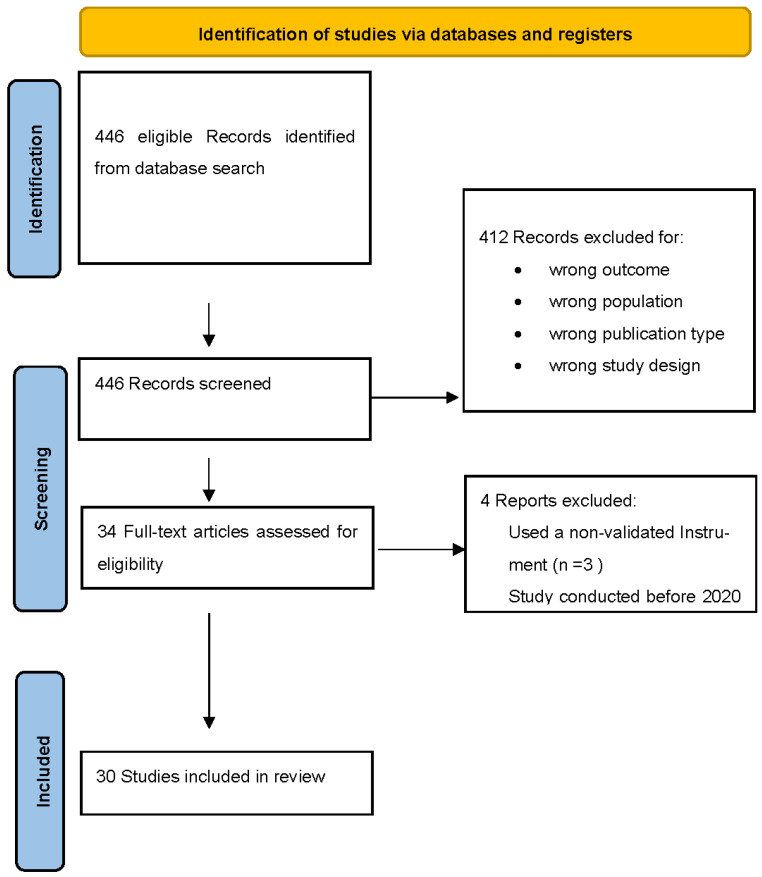
Study selection process. [25].

Our search strategy resulted in 446 possible eligible studies. The study investigators screened the titles and abstracts of these studies, resulting in 34 for probable inclusion and excluding 412 based on the predetermined inclusion criteria. The 34 remaining studies went through a full-text assessment for eligibility which resulted in 30 studies that were included in the final review and subsequent analysis. (Figure 1)

All included studies were cross-sectional studies assessing burnout at a single time point.

The geographic distribution of studies was as follows, twelve studies (40%) were from the American continents (USA, Canada, Brazil, and Ibero-American) [29,30,31,32,33,34,35,36,37,38,39,40], seven studies (23.3%) were from Asian countries (Turkey, Saudi Arabia, and Pakistan) [41,42,43,44,45,46,47], while other seven (23.3%) reported results from Europe (France, Portugal, Spain, Romania, Netherlands, and European Union countries) [48,49,50,51,52,53,54] as well as four (13.3%) were from the African continent (Egypt and Libya) [55,56,57,58]. The age range of physicians spanned from the early 20s to 70+ years, all studies included male and female participants.

The Studies sample consisted of practicing physicians and trainees from different specialties which were identified in some studies including Psychiatry, Emergency medicine, physiatrist, pediatricians, otolaryngologist, head and neck surgery, orthopedics, and primary care physicians, internist, intensivist, urologists, and neurosurgeons.

### 3.2. Measurement Tools for Burnout

Among the 30 included studies, four standardized questionnaires were used to measure burnout prevalence among physicians. The majority (22/30) reported burnout using MBI or one of its adapted or modified versions such as Abbreviated Maslach Burnout Inventory (aMBI) (3/30) [31,32,57], 2 single-item (MBI-22) (2/30) [34,35], MBI-HSS (7/30) [38,41,42,48,56], of them MBI HSS-MP variation was used in two studies [44,52]. One used the Dutch version of MBI -HSS (Utrecht Burnout Scale, UBOS) which has 20 items [51]. The variation of MBI was not identified in (9/30) of whom one study only reported the EE sub scale of MBI [55].

In total, four studies used Copenhagen Burnout Inventory (CBI) [39,40,43,49] one used the Portuguese version of CBI [49], two studies used single-item Mini-Z burnout assessment [29,37], while the other two used Professional Fulfillment Index to report similar outcomes [30,33].

### 3.3. Aggregate Prevalence of Burnout

**Figure 2:**
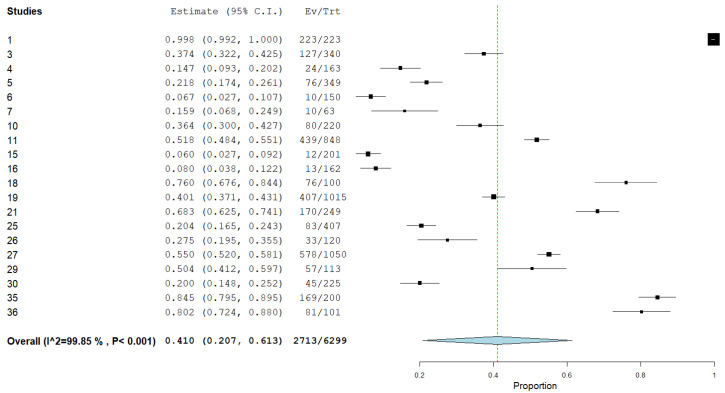
The aggregate prevalence of burnout.

A total of 6299 individual participants were included in the meta-analysis to calculate the aggregate prevalence of burnout (Figure 2). The aggregate prevalence of burnout was 41.0% (95% CI: 20.6–61.3%, I^2^ = 99.85%). The highest prevalence rate (99.8%) was reported by a study conducted on COVID-19 referral institutions in the State of Bahia, Brazil [36] which used the MBI tool. The lowest rate (6.0%) was conducted in Egypt which also used the MBI tool [58].

As noted above (i.e., I^2^ = 99.85%), we observed a high level of study heterogeneity on burnout. We conducted a meta-regression attempt to identify whether characteristics of the studies explained between-study variability in the burnout rates. From these analyses, only the burnout measures significantly explained study burnout variation (i.e., study location, physician category, and period of data collection were not significantly associated with burnout rates). Studies measuring burnout with the MBI showed the highest average burnout rates.

Out of the 30 total studies included in the systematic review, 10 studies were not included in the meta analysis due to missing data in which total burnout rates could be calculated. 

The geographic distribution of these 10 studies was as follows: 3 of the studies came from the United States, one from Ibero-America, two from Pakistan, two from Turkey, and one from Portugal and Libya. 

## 4. Discussion

Throughout the current COVID-19 pandemic, several studies have assessed the impact of COVID-19 on physician burnout, but the results have been inconsistent. One study using the Maslach Burnout Inventory (MBI) found that a significant number of physicians suffered from increased levels of burnout after the inception of COVID-19 [1]. However, another survey using the ProQOL Questionnaire showed that burnout levels among healthcare professionals remained similar to previous studies despite the COVID-19 health crisis [59].

Even in a particularly problematic COVID region, such as Italy, the results are nearly contradictory. Italy, one of the first western countries to be affected by the pandemic, had seen an alarming increase in the number of patients who had recovered and died; thus, the workload and demand for healthcare professionals had increased. In one study, which included a sample of 532 Italian physicians and other healthcare workers (HWs), the subjects answered an online MBI that assessed their burnout levels and frequency of psychosomatic symptoms experienced. The results showed that burnout levels and symptoms were associated with increased demand due to the COVID-19 epidemic [2]. 

Another Italian study compared the mental health status of physicians and other HWs before and during the COVID-19 crisis and found that clinically related symptoms of depression, anxiety, and burnout were more common in HWs during the COVID-19 epidemic than before the emergency [3]. In contrast, a study aimed at analyzing burnout levels among members of the Italian Association of Medical Endocrinologists (AME) before and during the COVID-19 outbreak found that the epidemic itself did not lead to changes in burnout levels, and short-term exposure to pandemic-related activities appeared to have little effect on the severity of burnout, except for physicians directly involved in the management of COVID-19 cases [60].

The 2021 Medscape National Physician Burnout Report revealed a burnout rate that was similar to the burnout rate reported in 2020 and 2015. It should be noted there may be several different causes for the variance in results, such as the month the data were collected, the situation of the pandemic in each country at the time of data collection, and the method used to assess burnout.

From 446 search results in this systematic review, we selected 30 studies that included a total of 6299 individual participants. Across the studies, the prevalence of physician burnout rates ranged from 6.0–99.8%. This wide variation could be due to the heterogeneity among burnout definitions, different applied assessment tools, and even cultural factors. However, even when the same burnout tool was used, there was no consensus on the burnout rate. This lack of consensus produced wide variability in burnout prevalence rate reports and limits our ability to make reliable comparisons between studies [15]. 

While the MBI is the most widely used tool for assessing burnout, there is no consensus on how to score this research tool. For example, researchers use different MBI cutoff points to define burnout. Doulougeri et al. [4] identified five prominent approaches to defining burnout using the MBI tool in their review, and Rotenstein et al. [5] found more than 142 unique definitions of burnout and 47 among studies using the MBI tool. According to the definition used by the authors, this variation suggests that there is either an overestimation or underestimation of burnout among the research population. Therefore, tools for assessing burnout should be better protocoled to enable consistent methods of scoring and interpretation. In addition, a recent study by Barker et al. [6] has shown that cultural factors may play a role in burnout. 

Most studies have used the MBI to assess burnout or one of its sub scales [7], while some countries have their own version of the MBI, such as the Utrecht Burnout Scale (UBOS) [8]. However, there is still a lack of a standardized tool to assess the prevalence of burnout, as it is mainly a research domain topic, not a clinical diagnosis [9,10]. Other commonly used tools include the Copenhagen Burnout Inventory (CBI), Mini Z Burnout Survey, and Oldenburg Burnout Inventory (OBI). There is no agreement on how to measure workforce burnout on the issue of variable definition and cutoff point for each study, so there is a need to highlight the burnout definition when discussing the prevalence of burnout among different studies as highlighted by Chloe Hivar et al. Moreover, different tools assess different concepts of burnout. For instance, the CBI and OBI were developed to overcome limitations of the MBI, including a lack of theoretical concept and psychometric limitations [11,12].

Nevertheless, research on burnout during the era of the pandemic is accumulating. It is essential for future studies to consider that most of the available literature is based on cross-sectional studies; therefore, the effect of the pandemic cannot be ascertained, as burnout could be present in pre-pandemic times as suggested; not a direct effect of the pandemic. One solution is to establish a baseline burnout level in pre-pandemic times or events between successive waves of COVID-19. Some authors compared the prevalence of physician burnout during the pandemic to non-pandemic times, compared frontline workers to non-frontline workers, or compared burnout from COVID-19 to other pandemics such as SARS-CoV or MERS [13,14]. This comparison gives rise to the importance of conducting periodic burnout assessments in healthcare facilities to establish a baseline for burnout and to determine an appropriate measure for intervention and prevention. 

Additionally, most included studies presented the prevalence as binary options (i.e., having burnout or not), which might limit different interpretations in the case of changing cutoff points and using previous studies’ values as a baseline. For example, even if the physician was experiencing burnout, did the mean score increase or decrease? It is therefore prudent to include a burnout mean score when reporting burnout for each sub scale. An additional advantage is the ability to use proper tests such as t-tests and ANOVA. 

Another important point is to consider other factors whenever we are assessing burnout, as most studies lack relevant variables such as the presence of a psychiatric disorder, is a history of chronic disease, substance abuse, depression, anxiety, and stress scales, which have shown an association with burnout [15,16,17]; burnout leads to the aforementioned disorders. It is also important to consider other work-related factors, either risk or protective factors, such as job hours, on-call work, break times, compensation, job security, and the presence of social support, as these are known to contribute to burnout prevalence among healthcare workers [61].

There is an ongoing debate that burnout experienced by physicians might have been developing since medical school [19] and residency training [62]. Neglected burnout might lead to concurrent psychiatric disorders such as stress, insomnia, alcohol or substance abuse, or an impaired social life. Additionally, physical symptoms related to the musculoskeletal system and gastrointestinal tract, and even chronic diseases such as diabetes and cardiovascular diseases [25]. To control the burnout prevalence or symptoms, individual and organizational measures could be implemented, such as practicing a healthy lifestyle that includes exercise, a nutritious diet, sufficient rest, and family support [15,26,27].

It is crucial to raise awareness about physician burnout, the provision of psychological supports, and the development of skills needed to overcome burnout. The lack of such neutralizing measures may result in delayed diagnoses and long-term sequelae. Organizational efforts are focused on preventive measures such as clarifying job tasks, accommodating work schedules, decreasing work demands for groups that are vulnerable to burnout, increasing access to resources, encouraging mindfulness activities, and providing psychological support services for those in need. These measures should be implemented by policymakers at the level of the institution or organization [14,15,27]. Nevertheless, having well-structured training in clinical or psychological skills results in higher efficacy, thereby increasing self-competence and reducing occupational stress and burnout [28,29].

Moreover, attending to mental health issues, reducing the workload of HWs through adjustments to work shifts, reducing job-related stressors, and creating a healthy work environment may prevent or relieve burnout. Future large and multi-center studies on HWs of COVID-19 wards are necessary to identify the frequency, associated factors, and effective preventative strategies of this phenomenon.

### Limitations

This review had several limitations that need to be recognized, including a small number of included articles, inconsistent methodologies, and heterogeneity in reporting approaches. In addition, there are no comprehensive tools to measure events that occur outside the workplace. Also, other serious disorders are overlooked. Burnout assessments suggest that such triggers may be overlapped with other psychiatric disorders (depression, anxiety, etc.); therefore, it is difficult to understand exactly how plausible the burnout data are. Furthermore, this review does not define how burnout differs among physicians of different specialties. Another limitation of this study is the cultural factor that may be relevant to the results of the studies considered in this review, factors that not considered in studies that may provide important explanations for some results. Of note, the current review includes a study of dramatically different disaster phases conducted between 1 January 2020 and 1 September 2021. Different stages of the COVID-19 pandemic compared to specific study time points undoubtedly contribute to the heterogeneity of the data. Finally, the long-term effects of burnout among physicians during the COVID-19 pandemic remain unclear, as long-term data are not yet available. However, this review will bring us more knowledge about the universal phenomenon of physician fatigue, which has a significant impact on patient care, and the facts presented in this report should be taken into account when assessing and solving this global problem.

## 5. Conclusions

It is difficult to anticipate whether the recent effect of the pandemic is transient or lasting, but COVID-19 could be an opportunity for healthcare organizations to examine the system, benefit from the COVID-19 experience, and take an active role in alleviating worker burnout with respect to recognizing its effect, implementing policy focused on work-life balance, and providing mental health access to everyone affected.

## Data Availability

All data used in the review are publicly available.
